# Phosphatidylcholine Membrane Fusion Is pH-Dependent

**DOI:** 10.3390/ijms19051358

**Published:** 2018-05-03

**Authors:** Sergey A. Akimov, Michael A. Polynkin, Irene Jiménez-Munguía, Konstantin V. Pavlov, Oleg V. Batishchev

**Affiliations:** 1 Laboratory of Bioelectrochemistry, A.N. Frumkin Institute of Physical Chemistry and Electrochemistry, Russian Academy of Sciences, 31/4 Leninskiy Prospekt, 119071 Moscow, Russia; polynkin.michael@gmail.com (M.A.P.); olegbati@gmail.com (O.V.B.); 2 Department of Theoretical Physics and Quantum Technologies, National University of Science and Technology “MISiS”, 4 Leninskiy Prospekt, 119049 Moscow, Russia; 3 Department of Engineering of Technological Equipment, National University of Science and Technology “MISiS”, 4 Leninskiy Prospekt, 119049 Moscow, Russia; w0r3ss@gmail.com; 4 Laboratory of Electrophysiology, Federal Clinical Center of Physical-Chemical Medicine of FMBA, 1a Malaya Pirogovskaya Street, 119435 Moscow, Russia; qpavlov@mail.ru; 5 Department of Physics of Living Systems, Moscow Institute of Physics and Technology (State University), 9 Institutskiy Lane, 141700 Dolgoprudniy Moscow Region, Russia

**Keywords:** membrane fusion, enveloped virus, influenza, stalk, theory of elasticity, pH dependence

## Abstract

Membrane fusion mediates multiple vital processes in cell life. Specialized proteins mediate the fusion process, and a substantial part of their energy is used for topological rearrangement of the membrane lipid matrix. Therefore, the elastic parameters of lipid bilayers are of crucial importance for fusion processes and for determination of the energy barriers that have to be crossed for the process to take place. In the case of fusion of enveloped viruses (e.g., influenza) with endosomal membrane, the interacting membranes are in an acidic environment, which can affect the membrane’s mechanical properties. This factor is often neglected in the analysis of virus-induced membrane fusion. In the present work, we demonstrate that even for membranes composed of zwitterionic lipids, changes of the environmental pH in the physiologically relevant range of 4.0 to 7.5 can affect the rate of the membrane fusion notably. Using a continual model, we demonstrated that the key factor defining the height of the energy barrier is the spontaneous curvature of the lipid monolayer. Changes of this parameter are likely to be caused by rearrangements of the polar part of lipid molecules in response to changes of the pH of the aqueous solution bathing the membrane.

## 1. Introduction

**Figure 1:**
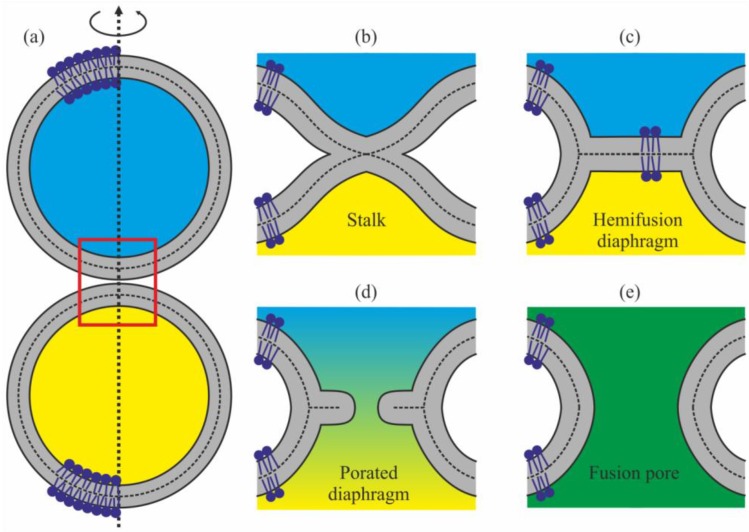
Schematics of a possible trajectory of the membrane fusion process. The membranes are shown in gray, the water volumes surrounded by them in blue and yellow. (**a**) Convergence of membranes with formation of tight contact. The contact location is designated by the red rectangle. (**b**) Stalk, a structure, in which the contact monolayers of membranes have already fused, while the distal ones have not yet done so. (**c**) Hemifusion diaphragm: during stalk expansion, lipids of the distal monolayers are brought into contact, thus forming a bilayer. (**d**) During diaphragm poration, the originally isolated aqueous compartments start mixing. (**e**) Fusion pore.

Fine rearrangements of membranes caused by local loss of integrity of two contacting membranes with subsequent connection of the affected membranes in a new topology continuously occur in nature. Such rearrangements, commonly known as fusion, result in uniting the aqueous interiors of cells, vesicles etc. without release of their contents into environment [1]. Membrane fusion underlies such processes as the formation of cellular membranes [2], cell proliferation and fertilization [3,4,5,6], exocytosis [7,8], vesicular transport and interaction of organelles inside the cells [9,10,11], cellular digestion with participation of lysosomes [12], synaptic transmission [13], virus infection [14] etc. The fusion of biological membranes is performed by special proteins or protein complexes, which change their conformation under the influence of certain triggers, performing work necessary to achieve tight contact between the lipid matrices of the membranes (Figure 1a). Thereafter, the fusion process mostly proceeds along the trajectory including formation of a structure known as the stalk [15], in which the contact monolayers have already fused, while the distal monolayers have not (Figure 1b). During stalk expansion, lipid molecules of the distal monolayers form “tail to tail” contact, thus forming a bilayer. This structure is known as a hemifusion diaphragm (Figure 1c) [16]. Pores can appear in the bilayer formed by lipids of distal monolayers of the fusing membrane (Figure 1d), leading to rupture of the hemifusion diaphragm and forming a fusion pore (Figure 1e) [17].

This scenario implies the continuity of lipid bilayer at all stages, in the sense that the water volumes surrounded by fusing membranes are never connected through a continuous pathway with the environment, i.e., the fusion is leakage-free (Figure 1). This fusion mechanism was experimentally demonstrated for a number of systems, including purely lipidic [16,18,19,20] and lipid-protein [21,22,23,24,25] systems. Although there are other possible ways of evolution of the system of fusing membranes, sometimes occurring with the rupture of one of the interacting membranes at the intermediate stage [26,27], it is the trajectory associated with radial expansion of intermediate stalk that proved to be highly efficient, whereas others do not result in complete fusion, i.e., they lead to a dead end [27]. In this respect, it is the lipid component of the biological membranes that plays an important role in fusion. The point is that the mechanical properties of the lipid matrix to a large degree determine the energy of intermediates, through which membrane fusion occurs, and therefore the elastic properties of the lipid bilayer determine the height of the intermediate energy barriers [28]. Presently, virus-induced fusion is the most clearly understood process, since in this case the entire process of bringing the membranes together is performed by a single fusion protein, i.e., hemagglutinin protein in the case of the influenza virus [29,30]. Influenza virus belongs to enveloped viruses (it has a protein shell covered with lipid membrane), and mostly penetrates into the cell by means of endocytosis [31,32]. The conformational rearrangement of hemagglutinin protein is triggered by a reduction of pH of the environment in a ripe endosome, causing the release of the fusion peptide from the hydrophobic pocket, its incorporation into the target membrane, and formation of a fusion rosette in which the proteins cooperatively bring the viral and endosomal membranes together [33] with the formation of tight contact. The investigation of the influence of the lipid component on the process actively continues to date [24,29,34,35].

Obviously, characteristics of membranes are inevitably dependent not only on their lipid composition but also on the external conditions, such as temperature, anion and cation composition, total ion strength, etc. One of the most important parameters is the environmental pH. Nevertheless, to the best of our knowledge, none of these publications addressed the influence of pH upon the elastic parameters of the lipid bilayer and molecular geometry of lipids, which defines the spontaneous curvature of lipid monolayers [24,29,34,35]. At the same time, the pH of the environment in the range of transition from neutral conditions (7.0–7.5) to the conditions of ripe endosomes (pH 4.5–5.0) can notably change the parameters of even zwitterionic lipids, such as phosphatidylcholine: membrane bending modulus, dipolar moment, structure of the polar part of the lipid molecule, and hence the spontaneous curvature of the monolayer [36,37,38]. In the existing models of membrane fusion based on the elastic properties of lipid bilayers, it has been shown that it is the bending modulus and spontaneous curvature that determine the elastic energy of fusion intermediates and the height of the energy barrier, defining the rate of the fusion process [17,28]. Thus, making allowance for the influence of pH upon the membrane fusion process would help in more accurately determining the mechanisms of operation of virus fusion proteins, as well as the continuous trajectory of the entire fusion process during virus-induced infection of cells.

In the present work, we used the model system consisting of two planar bilayer lipid membranes (BLM) formed by zwitterionic lipids to determine experimentally the rate of the initial stages of the fusion process as a function of pH. It was found that the fusion rate increases notably when the pH of the environment changes from 7.5 to 4.1. For the purpose of analyzing the obtained experimental results we developed a theoretical model of the initial stages of fusion of planar lipid membranes, yielding quantitative prediction of behavior of a system of interacting BLM.

## 2. Results

**Figure 2:**
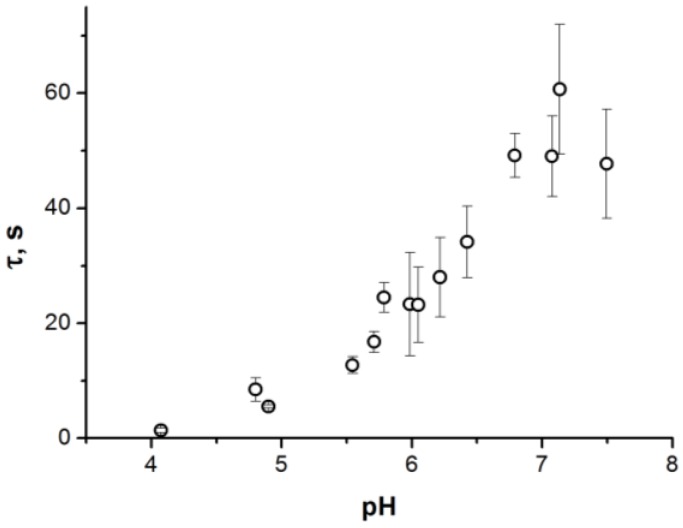
Dependence of the average waiting time for monolayer fusion of model lipid membranes upon pH of the solution. Each point represents a value averaged over 10 measurements. For points at pH = 4.1 and 4.9 the error bars are directed inside the representing circles, as the statistical error for these points is smaller than the size of the circle; thus, the circles appear as gray (partially filled).

We investigated experimentally the dependence of lag time to monolayer fusion for the membranes consisting of neutral zwitterionic lipids upon pH of the bathing solution in the physiologically relevant range of 4.1 to 7.5. We used the experimental system configuration suggested in [39], where bilayer lipid membranes of the desired composition were formed on orifices at the tips of Teflon cones, whereupon they were brought into tight contact and the fact of fusion of the contacting monolayers of the membranes was detected as an increase of the electric capacitance of the BLM system. Experiments with the dioleoylphosphatidylcholine (DOPC) membranes in a neutral environment (pH 7.0) indicated that the lag time for monolayer fusion exceeded two hours, which is comparable to lifetime of such membranes and greatly complicates obtaining meaningful results in such a system (data not shown). Therefore, we switched to the mixture of DOPC with dioleoylphosphatidylethanolamine (DOPE) 5:1 DOPC:DOPE, since DOPE is known to accelerate the process of monolayer fusion due to its molecular geometry [16], but still remains neutral in the investigated range of pH . In this system, maximal waiting times to monolayer fusion of the membranes were about one minute (Figure 2). As can be seen from the results of the experiments (Figure 2), pH increase from 4.1 to 7.5 results in substantial change of the rate of monolayer fusion: The average waiting time at the boundaries of the investigated range of pH differs approximately by a factor of 35, from ~1.4 s at pH = 4.1 to ~49 s at pH = 7.5 (Figure 2).

Assuming Arrhenius dependence of the lag time upon the energy barrier, as in [17]:

(1) $$\tau =\frac{1}{\nu }e^{\frac{E}{k_{B}T}}$$

where *E* is the energy barrier height; *k_B_*—Bolzmann constant; *T*—absolute temperature (*k_B_T* ~ 4⋅10^−21^ J); *ν*—characteristic frequency of attempts of the system to cross the energy barrier; the experimentally determined dependence of the average waiting time to fusion corresponds to change of the energy barrier approximately by 3.5 *k_B_T*. Indeed, if we designate the height of the barrier and the average waiting time at pH = 4.1 as *E*_4.1_ and *τ*_4.1_, respectively, and at pH = 7.5, as *E*_7.5_ and *τ*_7.5_, respectively, then:

(2) $$\frac{\tau_{7.5}}{\tau_{4.1}}=e^{\frac{E_{7.5}-E_{4.1}}{k_{B}T}}\approx 35$$

from which we can determine that *E*_7.5_ − *E*_4.1_ ≈ *k_B_T*ln(35) ≈ 3.5 *k_B_T*.

What could cause such a change of the energy barrier to fusion? As reported in [36,37], a decrease of environmental pH from 7 to 4 results in an increase of the membrane bending modulus by about 20%. It is intuitively clear that it should cause an increase, rather than a decrease of the fusion energy barrier, since growth of the bending modulus should result in an approximately proportional increase of the membrane deformation energy. In [36], it was demonstrated that a change of pH in this range also changes dipole potential of the membrane, which implies reorientation (turn) of lipid dipoles. Such a reorientation can, in principle, result in variation of the effective volume of lipid polar groups which, in turn, defines the spontaneous curvature of the lipid monolayer. Another potentially relevant factor is a change of the surface charge density of BLM with such lipid composition, as was observed in electrokinetic experiments [36,40]. However, assessment of the possible electrostatic contribution into the membrane interaction energy according to the Derjaguin–Landau–Verwey–Overbeek theory shows that the increase of ζ-potential observed for phosphatidylcholines in the neutral environment [36,40] yields, in our conditions, the electrostatic interaction energy of about 0.1 *k_B_T*, which clearly cannot explain the observed experimental dependence on the environmental pH . Thus, it would appear that change of pH should primarily modify the bending modulus [36,37] and spontaneous curvature of lipid monolayers.

**Figure 3:**
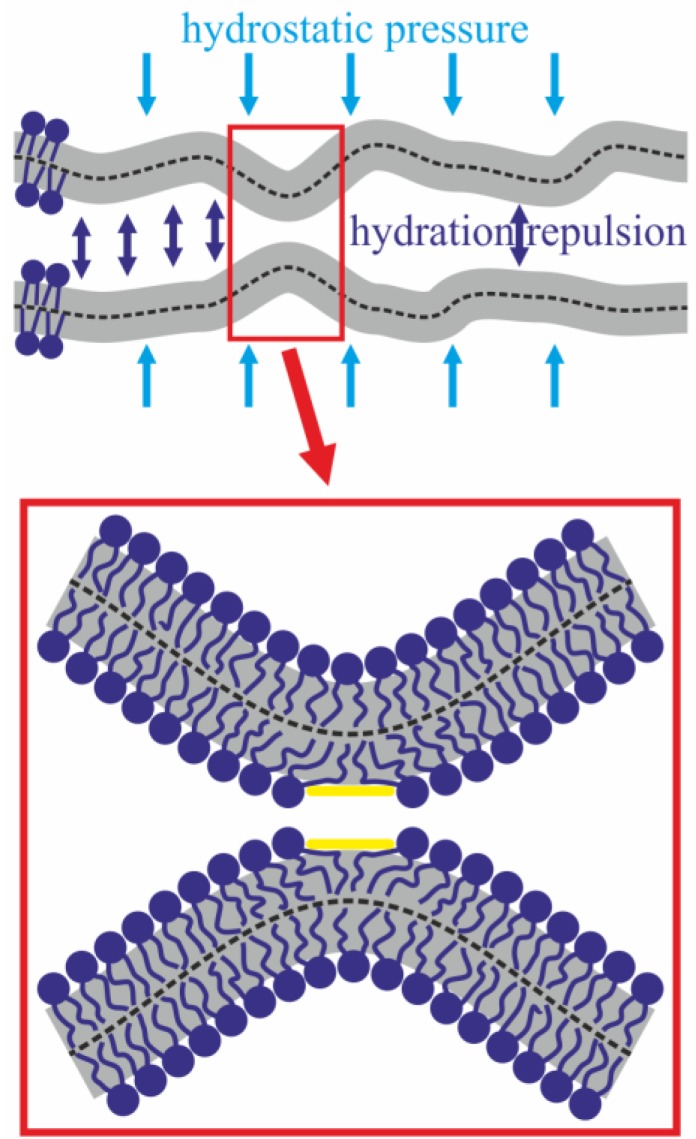
Top—Bilayer lipid membranes thermally fluctuate around the equilibrium distance between the monolayers, determined by the applied hydrostatic pressure and hydration repulsion. On the fluctuation-induced bulges facing each other (surrounded by red rectangle), lipids can displace laterally with the formation of hydrophobic spots (shown in yellow) at the expense of the hydration-induced repulsion.

In order to describe the process of membrane fusion at different pH values, we suggested a model of the process taking into account membrane deformation energy, hydration repulsion of the membranes, and attraction of the hydrophobic spots formed in the contact monolayers. It was assumed that when the membranes approach the distance of ~4–6 nm, the hydrostatic pressure necessary for the initial bulging of the membranes is equilibrated by the hydration repulsion forces (Figure 3, top).

It was assumed that the shapes of the opposing membrane leaflets can form bulges facing each other at the expense of thermal fluctuations [41]. On the tops of the bulges, lateral displacement of polar lipid heads from the contact area, resulting in the formation of hydrophobic spots, is possible due to powerful hydration-induced repulsion (Figure 3, bottom). Mutual attraction of the hydrophobic spots provides a driving force for fusion of the contact monolayers with formation of a stalk. It was assumed that the pH of the environment affects the magnitudes of the bending modulus and the spontaneous curvature of the lipids.

**Figure 4:**
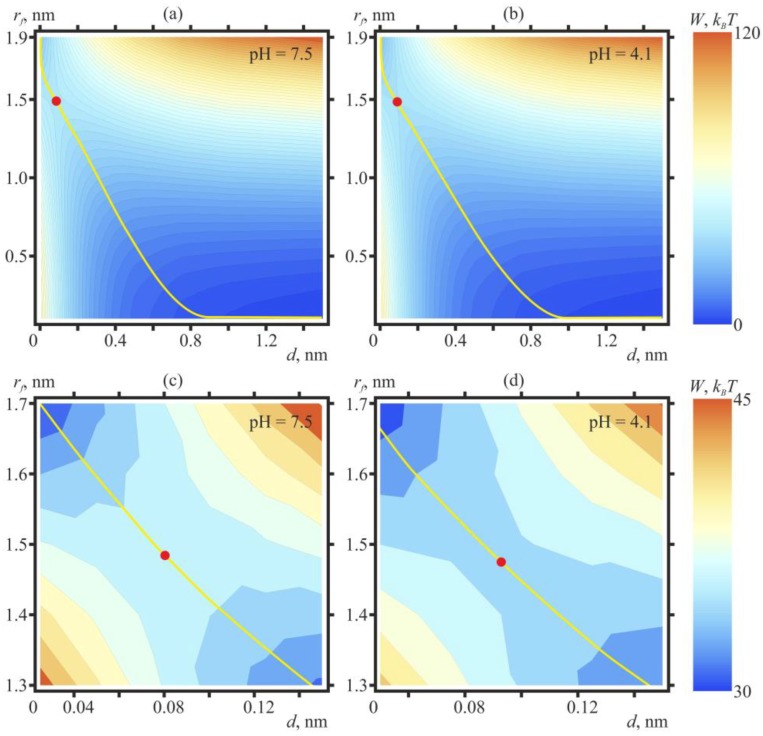
Contour plot (equal energy lines) of *W*(*r_f_*, *d*), calculated for dioleoylphosphatidylcholine (DOPC):dioleoylphosphatidylethanolamine (DOPE) = 5:1 membranes at pH = 7.5 (**a**,**c**) and at pH = 4.1 (**b**,**d**). For plots (**a**,**b**) blue color corresponds to 0, red—to 120 *k_B_T*; the distance between the isolines is 2 *k_B_T*. Red circles outline the saddle points of the energy surface, defining the heights of the energy barriers along the optimal trajectories of the fusion process (shown by yellow lines). The energy in the saddle points amounts to the following values: at pH = 7.5 (**a**)—39.5 *k_B_T*; at pH = 4.1 (**b**)—36 *k_B_T*. The plot (**c**) presents the zoomed vicinity of the saddle point of the plot (**a**) (pH = 7.5); the plot (**d**)—vicinity of the saddle point of the plot (**b**) (pH = 4.1). For plots (**c**,**d**) blue color corresponds to 30 *k_B_T*, red—to 45 *k_B_T*.

Our theoretical model of the process of fusion of planar lipid membranes allows calculation of the system energy, *W*(*r_f_*, *d*), as a function of half-distance between the bulges occurring on the membranes in the fluctuation mode, *d*, and the radius of the hydrophobic spots, *r_f_*, occurring on the surfaces of the bulges due to lateral displacement of the polar lipid heads from the area of tight contact between the monolayers. Figure 4 shows isolines of energy *W*(*r_f_*, *d*), calculated for DOPC:DOPE = 5:1 membranes at pH = 7.5 (Figure 4a,c) and at pH = 4.1 (Figure 4b,d). The energy is minimal at large distances between the membranes and zero radius of the hydrophobic spot, which corresponds to the equilibrium state of the membranes brought together by hydrostatic pressure. Besides that, when the distance between the membranes is small, and the radius of the hydrophobic spot is large, the energy is also minimal because of hydrophobic attraction between the spots. The energy grows abruptly at large *d* and large *r_f_* (hydrophobic surfaces are exposed into the bulk water on a large surface area), as well as at small *d* and smal *r_f_* (the hydration-induced repulsion of the membrane closely juxtaposed on a large area) (Figure 4).

Accordingly, there is a saddle point with the coordinates {*r_f_^s^*, *d^s^*} on the energy surface, determined by the following conditions:

(3) $$\frac{\partial W\left(r_{f}^{s},d^{s}\right)}{\partial r_{f}}=\frac{\partial W\left(r_{f}^{s},d^{s}\right)}{\partial d}=0$$

The energy in the saddle point determines the height of the energy barrier on the optimal trajectory of the fusion process. At pH = 7.5, the calculated value of the energy barrier amounts to *E*_7.5_ = 39.5 *k_B_T*; at pH = 4.1—*E*_4.1_ = 36 *k_B_T*, and thus, *E*_7.5_ − *E*_4.1_ = 3.5 *k_B_T*, in good agreement with the dependence of the average lag time of the monolayer fusion upon pH probed experimentally Equation (2), Figure 2. According to the estimates made in [17], the energy barrier of the height of ~40 *k_B_T* is to be crossed at the expense of thermal fluctuations of lipids over time of the order of 1 min, which is in good agreement with the measured average waiting time for monolayer fusion at pH = 7.5.

In the course of the calculation of the energy surfaces represented on Figure 4, we assumed that when pH changes from 7.5 to 4.1, the bending modulus and spontaneous curvature change by 20%. The bending modulus change was determined experimentally in [36,37]. There is but circumstantial evidence of changes of spontaneous curvature: when pH is varied, the dipole potential of the membrane changes considerably [36]. We also performed calculations under the assumption that pH variation between 7.5 and 4.1 increases the bending module by 20% whereas the spontaneous curvature of monolayers remains intact. In this case, the energy surfaces calculated at pH = 7.5 and pH = 4.1 virtually coincide, and the energy barrier height at pH = 4.1 amounts to 40 *k_B_T* (data not shown; the energy surface is visually almost indistinguishable from that shown in Figure 4a,c). Thus, increase of the bending modulus by 20% at the constant spontaneous curvature of lipid monolayers insignificantly affects the height of the energy barrier and the trajectory of the fusion process.

For the membranes consisting of pure DOPC, the calculation of the energy surface *W*(*r_f_*, *d*), performed for the elastic parameters corresponding to DOPC at pH = 7.5 revealed that the energy in the saddle point equals *E_DOPC_* = 44.5 *k_B_T*, and hence the average lag time for the membranes consisting of DOPC has to amount to:

(4) $$\tau_{DOPC}=\tau_{7.5}e^{\frac{\Delta E_{DOPC}-\Delta E_{7.5}}{k_{B}T}}=\tau_{7.5}e^{5}\approx \;150\;\min$$

in perfect agreement with the experimental observations.

## 3. Discussion

Earlier attempts to investigate the molecular mechanisms of membrane fusion revealed that the lipid component of cellular membranes can notably affect fusion process [24,29,34,35]. For example, in [42] the authors show that the fluidity of cell membranes strongly affects all the processes of their topological rearrangements. In particular, polyunsaturated lipids can increase the rate of fusion reactions. Fusion involves topological rearrangement of the interacting membranes; therefore, lipid bilayers in this process are exposed to significant elastic deformations. In terms of membrane elasticity, polyunsaturated lipids have lower bending modulus compared to saturated lipids and more negative spontaneous curvature [43]. Both factors facilitate fusion [16,17].

Fusion proteins and protein complexes enabling membrane fusion in various processes of the cellular lifecycle provide the energy needed for fusion [13,14]. However, direct measurement of the forces applied by fusion proteins, or accurate determination of the energy released during their conformational rearrangements, are almost never possible, and estimates are mostly based on circumstantial evidence. In many systems, these values are determined by evaluation of the work the proteins have to perform in order to induce the deformation of the interacting lipid membranes. Thus, it appears obvious that for accurately describing the molecular mechanism of membrane fusion processes, all the factors affecting elastic parameters of the membranes need to be taken into account. In this regard, in our opinion insufficient attention has been paid to the investigation of the properties of the environment (pH, ion strength) upon the membrane fusion process progression. However, even in such thoroughly investigated processes as fusion of enveloped viruses with endosomal membrane, the influence of an acidic environment of the late endosomes on the interacting membranes can affect the elastic parameters of membranes and the energy cost of formation of fusion intermediates. For the case of influenza virus, the pH drop inside the endosome with the trapped virus triggers the conformational rearrangement of hemagglutinin, the fusion protein of the virus, leading to anchoring of the target membrane and its subsequent fusion with the viral one [14]. The energy required for such a process directly depends on the mechanical properties of the lipid matrix of interacting membranes. Therefore, a change in membrane bending rigidity or spontaneous curvature of the lipid monolayer with pH allows for a re-estimation of the energetics of viral-induced fusion and the amount of fusion proteins in the fusion rosette necessary for successful fusion.

The ionic strength and membrane structure can also affect the rate of membrane fusion. Cell membranes are rather complex structures comprising dozens of lipid species even by headgroup. For the charged lipids, specific adsorption of counter-ions can shift the pK and change the overall membrane electrostatics [44]. In the case of low ionic strength the electrostatic repulsion between equally charged surfaces may influence the fusion process as well, according to the Gouy–Chapman theory. However, in the present work we explicitly took zwitterionic lipids with pK values far enough from the studied region of pH and very low surface potential values [36], excluding the possible effect of pK shift upon adsorption of monovalent ions. Moreover, estimations for the contribution of electrostatic repulsion into the energetics of fusion in our case show that even in such low ionic strength as 1 mM of KCl, the electrostatic energy will be around 1 *k_B_T*, and that is still much lower than the observed effect of pH change on the membrane mechanics.

Despite the zwitterionic lipids, phosphatidylcholines and phosphatidylethanolamines, being electrically neutral in the physiologically relevant range of pH (4 to 7.5), the elastic properties of membranes made from these lipids can change in response to pH variation [36,37]. However, the increase of the membrane bending modulus in the acidic environment observed in these works is expected to decelerate rather than accelerate the topological rearrangement of the membranes from the planar state into the stalk, which should have increased the lag time of the monolayer fusion. Our experimental data, on the other hand, are indicative of the opposite. Nonetheless, in [36], the pH-induced change of membrane dipole potential was observed. In the absence of dissociation of polar groups of zwitterionic lipids in the investigated pH range, we can state that the magnitude of the dipole moment of the polar head of the lipid is preserved, and its projection on the normal to the membrane plane changes, indicating its possible reconstruction [38]. In this case, an increase of the dipole potential in acidic environment is indicative of increased projection on the normal to the membrane plane, and hence of the decreased volume of the polar part of the lipid molecule. As a result of such a process, the spontaneous curvature of the monolayer would become more negative, which should accelerate the initial stage of membrane fusion [16]. In the framework of our elastic model, we demonstrated that a decrease of the lag time of the monolayer fusion of the membranes in the acidic environment observed in the experiment is achieved when the spontaneous curvature changes by 20%, and this parameter is a definitive factor determining the change of the fusion energy barrier. 

In our experimental system, we limited ourselves by the range of pH from 4.1 to 7.5. In a more acidic environment it is natural to expect neutralization of DOPC phosphates (pK ~ 2 [45]), leading to repulsion between lipid headgroups due to positively charged choline moieties. The repulsion should result in more positive spontaneous curvature of lipid monolayers, which is expected to drop the rate of monolayer fusion [1], although the bending modulus somewhat decreases in very acidic pH [36]. In more basic pH > 7.5 the bending modulus increases and at pH = 9 becomes approximately equal to the bending modulus at pH = 4.1 [36]. However, in range 6.5 < pH < 9 the monolayer spontaneous curvature remains almost constant, as the dipole potential of the membrane does not change [36]. As we demonstrated in the present work, the increase of bending modulus alone negligibly changes the energy barrier of monolayer fusion. Therefore, there is no reason to expect a substantial variation of the average waiting time for monolayer fusion at pH > 6.5. This reasoning is in agreement with our experimental observations: the average waiting time manifests saturation for pH > 6.7 (Figure 2).

To conclude, we have demonstrated, that rearrangement of the polar heads of the zwitterionic lipids under variable pH of the bathing solution can notably change the energy profile of lipid membrane fusion. This suggests a new approach to evaluation of the work to be performed by the fusion proteins to initiate membrane fusion.

## 4. Materials and Methods

### 4.1. Experimental Section

**Figure 5:**
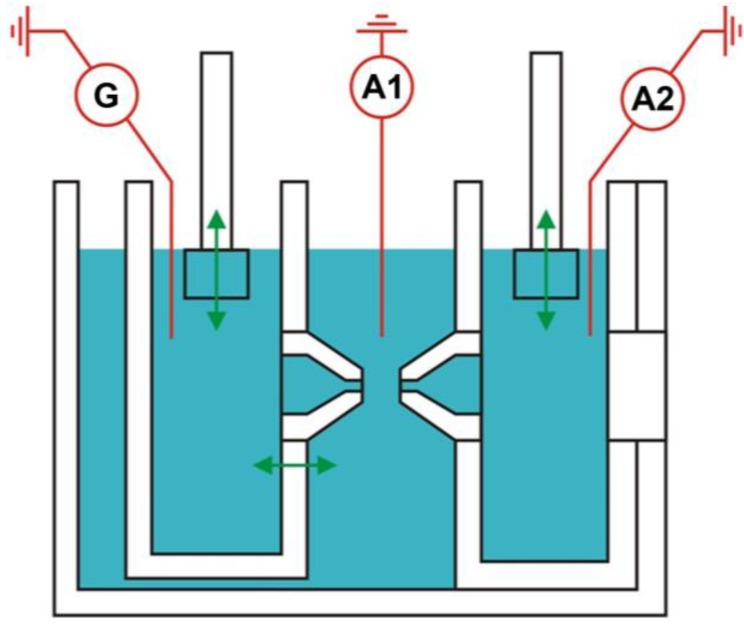
Schematic representation of the experimental cell used for investigation of the model bilayer lipid membranes. G is the generator; A1 and A2 are operational amplifiers. Green arrows represent the directions of the movement of the cell parts.

For investigation of the fusion process we used the experimental setup configuration suggested in [39] (Figure 5). Prior to the experiment, all the compartments of the Teflon cell were filled with a buffer solution of the following composition: 100 mM KCl, 5 mM HEPES in bidistilled water. The required pH value was achieved by adding an appropriate amount of HCl or KOH. An Ag/AgCl electrode was submerged in each of the three compartments of the cell. The bilayer lipid membranes were formed with the aid of the Muller–Rudin technique [46] from the solution of DOPC or (5:1) mixture of DOPC:DOPE (Avanti Polar Lipids, Alabaster, AL, USA) in decane (≥99.5%, Fluka Chemie, Buchs, Switzerland) (10 g/L) on 1 mm diameter orifices on the tips of Teflon cones in the central part of the cell (Figure 5). We used a mixture of lipids, since the membranes formed of pure DOPC brought in tight contact fail to fuse on the timescale of tens of minutes. Observation of the process of membrane formation was performed both optically, with the aid of two microscopes oriented in parallel and perpendicularly to the axes of the cones, and electrically, by means of measuring the electrical capacitance of the membrane. In order to determine the electrical capacitance of lipid membranes, electrical pulses with the amplitude of 100 mV (peak to peak) and frequency of 100 Hz were applied from the functional generator (model GFG-8219A, GW Instek, New Taipei City, Taiwan). Current responses were recorded with the aid of two operational amplifiers (model Keithley 427, Keithley Instruments, Solon, OH, USA), for each of the membranes respectively, and used for calculation of their electric capacitance. After forming a lipid bilayer on each of the cones, hydrostatic pressure of *P_app_* ~ 10 Pa was applied between the peripheral and the central compartments of the cell by means of plunging Teflon pistons into the peripheral compartments in order to form bubbles facing each other. Thereafter, the tips of the cones were brought in tight plane–parallel contact. According to [16], in order to reproduce the initial conditions, we maintained the diameter of the BLM contact area in the range of 0.25–0.35 mm. The parameter measured in the experiment was the monolayer fusion lag time, i.e., the time between bringing the membranes into plane–parallel contact and the time when the hemifusion diaphragm was formed. Fusion was detected as an abrupt increase of the observed electrical capacitance of the contact area. 

### 4.2. Theoretical Section

The process of fusion imposes considerable deformations on the participating membranes, associated with the topological rearrangements of the interacting membranes. In order to calculate the elastic energy, liquid crystal elasticity theory adapted to lipid membranes was used [47]. In the framework of this theory, the average orientation of lipid molecules is determined by a field of unit vectors, commonly known as directors, **n**. This field is defined on a certain surface inside the lipid monolayer, known as the dividing surface. The shape of the dividing surface is determined by the field of unit normals to it, **N**. We took credit for the following deformations of the membrane: (1) bending, characterized by divergence of the director along the dividing surface, div(**n**); (2) tilt, characterized by deviation of the director from the normal, (**n**–**N**); (3) lateral compression/expansion, characterized by the local change of the area of the dividing surface, *α* = (*a* − *a*_0_)/*a*_0_, where *a* and *a*_0_—are the current and the initial area of the dividing surface; (4) saddle-like bending, in a certain Cartesian system of coordinates {*x*, *y*} defined as $K=\frac{\partial n_{x}}{\partial x}\frac{\partial n_{y}}{\partial y}-\frac{\partial n_{x}}{\partial y}\frac{\partial n_{y}}{\partial x}$, where *n_x_*, *n_y_* are the respective projections of the director. Besides, we took into account that Muller–Rudin membranes are connected to a lipid reservoir, and hence certain lateral tension, 2*σ*_0_, is inevitably present. The neutral surface, where by definition the bending and lateral tension/compression deformations are independent of each other in terms of energy, was used as a dividing surface. It was experimentally determined that such a surface lies near the area of junction of polar heads and carbohydrate tails of the lipids at the depth of ~0.7 nm from the outer polar surface of the monolayer [48]. The deformations were assumed to be small, and hence the energy was calculated to the second order of deformations:

(5) $$W=\int \;\left(\frac{B}{2}{\left(\mathrm{div}n+J_{0}\right)}^{2}-\frac{B}{2}J_{0}^{2}+\frac{K_{t}}{2}{\left(n-N\right)}^{2}+\frac{K_{a}}{2}\alpha^{2}+\sigma_{0}+K_{G}K\right)dS-\sigma_{0}A_{0}$$

In this expression, *B*, *K_t_*, *K_a_*, *K_G_* are the moduli of bending, tilt, lateral stretch/compression, saddle-like bending, respectively; *J*_0_—spontaneous curvature of lipid monolayer; integration is performed over the neutral surface of the monolayer; *A*_0_—initial area of the neutral surface of non-deformed monolayer. It is assumed that the bulges formed through fluctuations on the juxtaposed membranes have local polar symmetry. Let us introduce a polar coordinate system *Ozr*, origin *O* and axis *Or* of which are located in the neutral surface plane of the distal monolayer of one of the membranes, the lower one, for the sake of precision, the *Oz* axis coincides with the axis of rotational symmetry of the system. Due to polar symmetry, all the deformations depend exclusively on the coordinate *r*, i.e., the system is unidimensional. Thus, all the vectors can be replaced with their projections on the *Or* axis: **n** → *n_r_* = *n*, **N** → *N_r_* = *N*, whereas divergence of the director, with the adopted degree of accuracy, equals div(**n**) = *n*′ + *n*/*r*, where prime sign stands for derivative by *r*. Hereafter, we call the projection of the director and normal upon *Or* simply “director” and “normal”. The hydrophobic part of the lipid bilayer can be considered volumetrically incompressible [49,50], i.e., the volume of the monolayer element is not affected by deformations. The local incompressibility condition can be written as [47]:

(6) $$h_{c}=h-\frac{h^{2}}{2}\mathrm{div}n-h\alpha$$

where *h_c_*—is the current thickness of the monolayer, *h*—monolayer thickness in the undeformed state. The values related to the upper (contact) and the lower (distal) monolayer will be designated by “*u*” and “*d*” indexes, respectively. The state of each membrane is characterized by seven functions: (1) directors of the upper (contact) and the lower (distal) monolayers, *n_u_*(*r*) and *n_d_*(*r*); (2) relative changes of the area of neutral surfaces of monolayers, *α_u_*(*r*) and *α_d_*(*r*); (3) distance from the *Or* plane to the intermonolayer surface, *m*(*r*); (4) distance from the *Or* plane to the neutral surface of the upper and the lower monolayers, *H_u_*(*r*) and *H_d_*(*r*). In this notation, the local incompressibility condition, Equation (6), reads as follows:

(7) $$\begin{array}{l} H_{u}-m=h-\frac{h^{2}}{2}\left(n_{u}^{\prime }+\frac{n_{u}}{r}\right)-h\alpha_{u}\\ m-H_{d}=h-\frac{h^{2}}{2}\left(n_{d}^{\prime }+\frac{n_{d}}{r}\right)-h\alpha_{d} \end{array}$$

With the required accuracy, *N_u_* = *H*′*_u_*(*r*), *N_d_* = −*H*′*_d_*(*r*), and:

(8) $$\int \sigma_{0}dS-\sigma_{0}A_{0}=\int 2\pi r\sigma_{0}\left(\sqrt{1+{\left(H_{u,d}^{\prime }\right)}^{2}}-1\right)dr\approx \int \pi r\sigma_{0}{\left(H_{u,d}^{\prime }\right)}^{2}dr$$

for the upper and the lower monolayer, respectively. After substitution, the total elastic energy functional, Equation (5), reads as follows:

(9) $$\begin{array}{l} W=\int_{R_{1}}^{R_{2}}2\pi rdr\frac{K_{t}}{2}(l^{2}{\left(n_{u}^{\prime }+\frac{n_{u}}{r}+J_{0}\right)}^{2}-l^{2}J_{0}^{2}+{\left(n_{u}-m^{\prime }+\frac{h^{2}}{2}\left(n_{u}^{\prime \prime }+\frac{n_{u}^{\prime }}{r}-\frac{n_{u}}{r^{2}}\right)+h\alpha_{u}^{\prime }\right)}^{2}+A\alpha_{u}^{2}+\\ +\sigma {\left(m^{\prime }-\frac{h^{2}}{2}\left(n_{u}^{\prime \prime }+\frac{n_{u}^{\prime }}{r}-\frac{n_{u}}{r^{2}}\right)-h\alpha_{u}^{\prime }\right)}^{2}+k_{G}n_{u}^{\prime }\frac{n_{u}}{r})+\int_{R_{1}}^{R_{2}}2\pi rdr\frac{K_{t}}{2}(l^{2}{\left(n_{d}^{\prime }+\frac{n_{d}}{r}+J_{0}\right)}^{2}-l^{2}J_{0}^{2}+\\ +{\left(n_{d}+m^{\prime }+\frac{h^{2}}{2}\left(n_{d}^{\prime \prime }+\frac{n_{d}^{\prime }}{r}-\frac{n_{d}}{r^{2}}\right)+h\alpha_{d}^{\prime }\right)}^{2}+A\alpha_{d}^{2}+\sigma {\left(m^{\prime }+\frac{h^{2}}{2}\left(n_{d}^{\prime \prime }+\frac{n_{d}^{\prime }}{r}-\frac{n_{d}}{r^{2}}\right)+h\alpha_{d}^{\prime }\right)}^{2}+k_{G}n_{d}^{\prime }\frac{n_{d}}{r}) \end{array}$$

where $l=\sqrt{B/K_{t}}$, *A* = *K_a_*/*K_t_*, *σ* = *σ*_0_/*K_t_*, *k_G_* = 2*K_G_*/*K_t_*. The first integral is taken over the neutral surface of the upper monolayer, the second over the neutral surface of the lower monolayer. The functional variation, Equation (9), with respect to the functions *n_u_*, *n_d_*, *m*, *α_u_*, *α_d_*, yields five Euler-Lagrange differential equations. The equations are quite cumbersome, and therefore not presented here. However, they can be solved analytically, the general solution of the system being as follows:

(10) $$\begin{array}{l} n_{u,d}=\pm \frac{c_{1}}{r}\pm c_{2}I_{1}\left(\sqrt{\frac{\sigma }{1+\sigma }}\frac{r}{l}\right)\pm c_{3}K_{1}\left(\sqrt{\frac{\sigma }{1+\sigma }}\frac{r}{l}\right)+\\ +c_{4}Y_{1}\left(p_{1}r\right)+c_{5}J_{1}\left(p_{1}r\right)+c_{6}Y_{1}\left(p_{2}r\right)+c_{7}J_{1}\left(p_{2}r\right) \end{array}$$

where the upper signs (+/–) relate to *n_u_*, the lower—to *n_d_*, and

(11) $$p_{1,2}=\sqrt{\frac{2A\left(h^{2}-l^{2}\right)-2\sigma h^{2}\pm 2\sqrt{\sigma^{2}h^{4}+A^{2}\left(l^{4}-\sigma h^{4}-2l^{2}h^{2}\right)-2h^{2}A\left(2l^{2}+\sigma \left(h^{2}+l^{2}\right)\right)}}{h^{2}\left(4l^{2}+h^{2}A\right)\left(1+\sigma \right)}}$$

Relative change of the neutral surface area is:

(12) $$\begin{array}{l} \alpha_{u}=\alpha_{d}=\frac{2+2l^{2}p_{1}^{2}-h^{2}p_{1}^{2}}{p_{1}h\left(2+A\right)}\left(c_{4}Y_{0}\left(p_{1}r\right)+c_{5}J_{0}\left(p_{1}r\right)\right)+\\ +\frac{2+2l^{2}p_{2}^{2}-h^{2}p_{2}^{2}}{p_{2}h\left(2+A\right)}\left(c_{6}Y_{0}\left(p_{2}r\right)+c_{7}J_{0}\left(p_{2}r\right)\right) \end{array}$$

the shape of the intermonolayer surface is written as:

(13) $$m=c_{1}\ln\left(r\right)+c_{2}\frac{\sigma h^{2}+2l^{2}}{2l\sqrt{\sigma \left(1+\sigma \right)}}I_{0}\left(\sqrt{\frac{\sigma }{1+\sigma }}\frac{r}{l}\right)-c_{3}\frac{\sigma h^{2}+2l^{2}}{2l\sqrt{\sigma \left(1+\sigma \right)}}K_{0}\left(\sqrt{\frac{\sigma }{1+\sigma }}\frac{r}{l}\right)$$

In the expressions (10)–(13) *I*_0_, *I*_1_, *K*_0_, *K*_1_, *Y*_0_, *Y*_1_, *J*_0_, *J*_1_ are the respective Bessel functions—of order zero and one; *c*_1_, *c*_2_, ..., *c*_7_—complex coefficients, which need to be determined from the boundary conditions. We imposed on the solutions (10)–(13) of Euler–Lagrange equations the following boundary conditions: (1) all the functions must be real for any real argument *r*; (2) all the functions must be limited for any *r*; (3) the director is continuous everywhere; (4) neutral surfaces of monolayers determined from Equation (7) are continuous everywhere besides the hydrophobic spots on the tops of the membrane bulges (Figure 3); (5) the director of contact monolayer on the boundary of the hydrophobic surface spot, *r* = *r_f_*, equals *n_u_*(*r_f_*) = −*r_f_*/*h*; (6) away from the bulges; the distance between the neutral surfaces of contact monolayers equals 2*Z*_0_ ≈ 6 nm, which is determined from the condition of equilibrium of the planar membranes brought into proximity by hydrostatic pressure *P_app_* ≈ 10 Pa; (7) distance between the neutral surfaces of contact monolayers at the boundary of the hydrophobic spot (*r* = *r_f_*) equals 2*d*. These conditions allow the determination of some of the coefficients; other free constants are determined by minimization of elastic energy. Ultimately, we obtained the free energy of the system as a function of two parameters: hydrophobic spot radius, *r_f_*, and half-distance between the hydrophobic spots in two membranes, *d*. The final expression for the free energy is extremely cumbersome and is omitted here.

Besides the membrane deformation energy, we also took into account the hydrophobic interactions and hydration repulsion between the fusing membranes. The energy of interaction between two planar hydrophobic spots in the contact monolayers separated by water interlay 2*d* is found as follows [51]:

(14) $$W_{f}=2\sigma_{W}\pi r_{f}^{2}\left(1-e^{-\frac{2d}{\xi_{f}}}\right)$$

where *σ_W_* is the macroscopic surface tension on the boundary between water and hydrocarbon chains of the lipids; *ξ_f_*—characteristic length of hydrophobic interactions. The energy associated with the hydration repulsion between the membranes is found according to the expression:

(15) $$W_{h}=P_{0}\xi_{h}\int e^{-\frac{z\left(r\right)}{\xi_{h}}}dS$$

where *z*(*r*) is the distance between the contact monolayers of the membranes at a given value of coordinate *r*; *ξ_h_*—characteristic length of hydration interaction; *P*_0_ is the wedging pressure determining the amplitude of hydration repulsion [52,53]; integration is performed over the hydrophilic surface of the contact monolayers. In order to qualitatively define the value of the integral in Equation (15), we use the Derjaguin–Landay–Verwey–Overbeek theory, according to which integration in Equation (15) can be restricted to the area, in which the distance between the membranes is increased by a value smaller than or equal to *ξ_h_*, having replaced the deformed hydrophilic surfaces of the contact monolayers on the horizontal planes.

In order to obtain quantitative results, the following parameter values were used: monolayer bending modulus *B* = 8 *k_B_T* [43]; tilt modulus (per monolayer) *K_t_* = 40 mN/m [47,54]; lateral stretch/compression modulus (per monolayer) *K_a_* = 100 mN/m [43]; saddle-like bending modulus (per monolayer) *K_G_* = −0.5*B* ≈ −4 *k_B_T* [55]; monolayer thickness *h* = 2 nm [43]; characteristic length of hydrophobic interactions *ξ_f_* = 1 nm [51]; surface tension of water/lipid hydrocarbon chains macroscopic boundary *σ_W_* = 40 mN/m [17,54]; characteristic length of hydration repulsion *ξ_h_* = 0.35 nm [53]; wedging pressure *P*_0_ = 6⋅10^8^ Pa [53], lateral tension of the membrane 2*σ*_0_ = 1.5 mN/m. Spontaneous curvature of monolayers was calculated as the spontaneous curvature of lipid components averaged with the weighing factors proportional to their molar concentrations. As was demonstrated earlier [56], additivity of the spontaneous curvature can be violated if saturated lipids are combined with cholesterol. We used lipid components with identical unsaturated (oleic) hydrocarbon chains; therefore there is no reason to consider the spontaneous curvature non-additive. It was assumed that at neutral pH the spontaneous curvature of DOPC *J_DOPC_* = −0.091 nm^−1^ [57]; for DOPE *J_DOPE_* = −0.399 nm^−1^ [57]; and for DOPC:DOPE = 5:1 mixture—*J*_0_ = 5/6*J_DOPC_* + 1/6*J_DOPE_* = −0.142 nm^−1^. We also assumed that change of pH from 7.5 to 4.1 results in a change of the bending modulus and spontaneous curvature of a monolayer by 20%, i.e., they become equal to 1.2*B* and 1.2*J*_0_, respectively.

## Abbreviations

BLM	Bilayer Lipid Membrane
DOPC	Dioleoylphosphatidylcholine
DOPE	Dioleoylphosphatidylethanolamine
